# Functional and molecular characterization of single serial killer CAR+ T cells demonstrates adaptive modification of behavior and fate based on tumor cell density

**DOI:** 10.1186/2051-1426-2-S3-P145

**Published:** 2014-11-06

**Authors:** Navin Varadarajan, Ivan Liadi, Gabrielle Romain, Harjeet Singh, Laurence Cooper

**Affiliations:** 1 University of Houston, TX, USA; 2 University of Texas M.D. Anderson Cancer Center, Houston, TX, USA

## 

**Figure 1:**
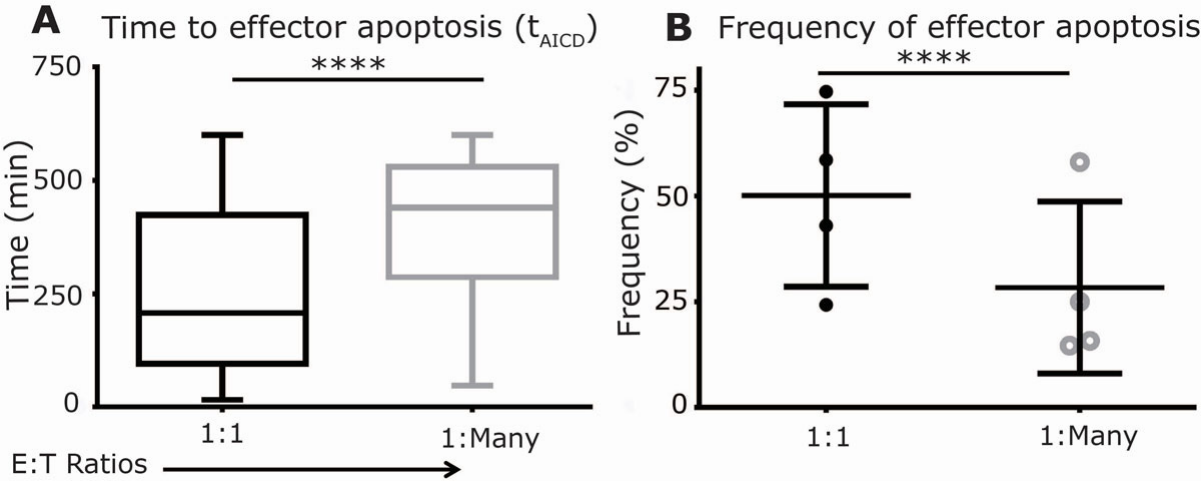
**Frequency and kinetics of effector apoptosis are dependent on functional activation by varying numbers of tumor cells**. (A) Box and whisker plots (extremities indicate range) of the distribution of single CAR^+ ^T cells undergoing AICD. (B) Donor variation in the average frequencies of apoptotic killer CAR^+ ^T cells. P-values were computed using an unpaired two-tailed t-test.

T cells genetically modified to express a CD19-specific chimeric antigen receptor (CAR) for the investigational treatment of B cell malignancies comprise a heterogeneous population, and their ability to persist and participate in serial killing of tumor cells is a predictor of therapeutic success. We developed Timelapse Imaging Microscopy In Nanowell Grids (TIMING) to dynamically analyze thousands of interactions between T cells and tumor cells. Our results demonstrate that while CAR^+ ^T cells launch fully-competent anti-tumor responses as defined by polarized motility, serial-killing and IFN-γ secretion, the cytolytic functionality of CAR^+ ^T cells must be quantified in the context of their ability to resist apoptosis. The ability of killer CAR^+ ^T cells to adaptively modify both cell-intrinsic (polarization and motility) and extrinsic (conjugation and kinetics of tumor-cell death) behavior was modulated by number of tumor cell encounters. By comparing both the serial killer and single killer CAR^+ ^T cells it appears that the propensity and kinetics of effector apoptosis was markedly different, effectors underwent apoptosis at higher frequencies (50%, single target killers, vs 28%, serial killers) and faster kinetics (time to apoptosis: 256 ± 12 minutes, single target; vs. 396 ± 25 minutes, multiple targets; p-value < 0.0001) (Figure 1). These data indicate that activation for lysis through multiple targets as opposed to prolonged contact with a single target reduces the propensity for effector apoptosis. Although the mechanistic basis for the responsiveness of these T cells to antigen/target density is not known, it is conceivable that the continuous propagation of these cells on irradiated aAPC at defined ratios, allows for balanced activation while minimizing AICD. These data could provide mechanistic insights into observations that infused CAR^+ ^T cells swell in number and produce high levels of cytokine in response to addressing large numbers of CD19^+ ^tumor cells, but then decline in number as the tumor bioburden is lowered due to the serial killing by effector T cells. Our findings emphasize the notion that motility and persistence (resistance to apoptosis), in addition to direct cytolytic activity, may serve as criteria to rapidly evaluate CAR^+ ^T cell potency. We anticipate that our findings and the TIMING methodology may provide a rapid approach for assessing the impact of CAR designs and sub-populations of cells modified to redirect specificity.

